# Exploration of laparoscopic day surgery mode for pediatric inguinal hernia: a large cohort study

**DOI:** 10.1007/s13304-025-02141-0

**Published:** 2025-03-08

**Authors:** Shuhao Zhang, Duote Cai, Yi Jin, Wenjuan Luo, Qingjiang Chen, Xiaoyan Fan, Zhigang Gao

**Affiliations:** Department Of General Surgery, Children’s Hospital, Zhejiang University School of Medicine, National Clinical Research Center for Children’s Health, Hangzhou, 310052 Zhejiang China

**Keywords:** Pediatric inguinal hernia, Day surgery, Laparoscopic repair

## Abstract

The pediatric day surgery has experienced a rapid development in recent years. This study aims to investigate the prospects of day surgery in pediatric hospitals. A total of 17,549 pediatric patients with inguinal hernia (IH) treated between July 2018 and August 2023 were included. The patients were divided into four groups: group A1 (open IH repair [OIHR] in the traditional ward), A2 (laparoscopic IH repair [LIHR] in the traditional ward), B1 (OIHR in the day ward), and B2 (LIHR in the day ward). A retrospective analysis was conducted based on clinical data, satisfaction and prognosis. The present study showed that the operative time between groups A2 and B2 showed no significant difference (*p* = 0.1205). However, the total time from entering to exiting the operating room was significantly longer in group A2 compared to group B2 (*p* < 0.0001). Hospitalization costs were significantly lower for patients in the day ward compared to the traditional ward (*p* < 0.0001). There was no significant difference in the recurrent IH rate between groups A2 and B2 (*p* = 0.977) or in incision infection rates between the day and traditional wards. The recurrent IH rate was significantly higher after OIHR compared to LIHR (*p* < 0.0001). The parent satisfaction in the day ward is higher than the traditional ward. The day surgery model of LIHR is a safe, reliable, and economically beneficial surgical management model that is highly recommended for pediatric hospitals.

## Introduction

Inguinal hernia (IH) is a common condition in pediatric patients, accounting for a significant portion of pediatric surgeries [1]. Traditional high ligation of the hernia sac is the standard procedure for inguinal hernia; however, postoperative complications, such as scrotal swelling, steep learning curves for young surgeons, and a high recurrent IH rate is associated with this approach [2, 3]. With the advent of minimally invasive techniques, laparoscopic surgery has transformed the management of pediatric IH, shifting from open to laparoscopic repair [4]. The first laparoscopic inguinal hernia repair (LIHR) was performed in 1993, and since then, the International Pediatric Endosurgery Group (IPEG) has favored laparoscopic repair owing to the shorter operative time and lower incidence of other complications, such as wound infections, hydroceles, and testicular atrophy [5]. LIHR now has become the main surgical method for pediatric Ihs [6].

Day surgery, first proposed by Scottish pediatric surgeon Nicoll in 1909, has gained rapid acceptance in recent years [7, 8]. Concurrently, with the rise of Enhanced Recovery After Surgery (ERAS), many medical centers have adopted day surgery practices [9]. These advanced management practices help reduce healthcare costs, shorten hospital stays, and improve patient satisfaction. In this context, pediatric LIHR has also gradually shifted from traditional inpatient surgery to day surgery. However, concerns about the safety of day surgery remain a major factor affecting parental acceptance and limiting its rapid expansion. Since 2016, our center has explored a day surgery model for pediatric IH repairs. This study analyzed medical data from our center to determine the safety, effectiveness, and socioeconomic benefits of day surgery for pediatric LIHR.

## Materials and Methods

### Patients and study design

Data from a total of 17,549 inguinal hernia patients who were treated at the Department of General Surgery or Department of Day Surgery from July 2018 to August 2023 were collected. All surgeries were performed by the same surgical team from the Department of General Surgery. Variables including sex, age, His classifications (unilateral of bilateral), surgical method (LIHR or OIHR), operative time, length of hospital stay, hospitalization cost, incision infection and recurrence rate of IH were recorded. A comparative analysis was conducted among group A1 (OIHR in traditional ward), group A2 (LIHR in traditional ward), group B1 (OIHR in day ward), group B2 (LIHR in day ward) based on the clinical data.

### Inclusion and exclusion criteria

The inclusion criteria were as follows: 1) children < 16 years of age; 2) children with an IH admitted to the Department of General Surgery or Day Ward at The Children's Hospital of Zhejiang University School of Medicine; and 3) absence of co-existing diseases.

The exclusion criteria were as follows: 1) children requiring emergency surgery due to incarcerated IH; 2) identification of a concurrent direct hernia during surgery; 3) simultaneous treatment of other diseases, such as phimosis, during IH repair; and 4) children < 3 months of age.

### Traditional ward inpatient process

**Fig. 1:**
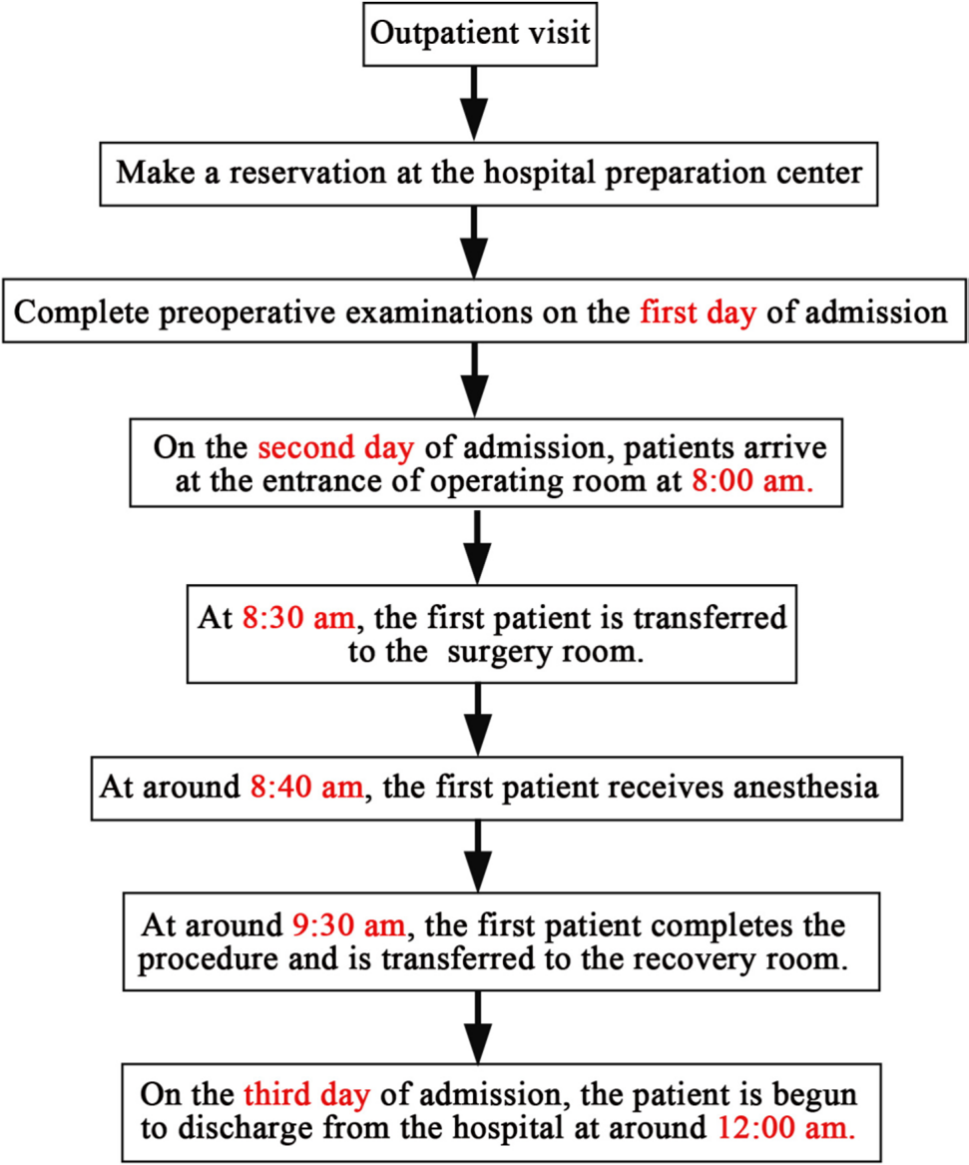
Process of traditional ward inpatient hospitalization

On the first day of admission, patients undergo all the preoperative examinations. Fasting begins at midnight, with water allowed from 6 am on the second hospital day. The first patient arrives at the operating room entrance at 8:00 am and is transferred into the operating room at 8:30 am. Endotracheal intubation anesthesia is typically administered at 8:40 am. The first procedure is completed around 9:30 am, after which the patient is moved to the recovery room for observation. The patient is discharged on the third hospital day at 12:00 am if no special conditions arise (Fig. 1).

### Day ward inpatient process

**Fig. 2:**
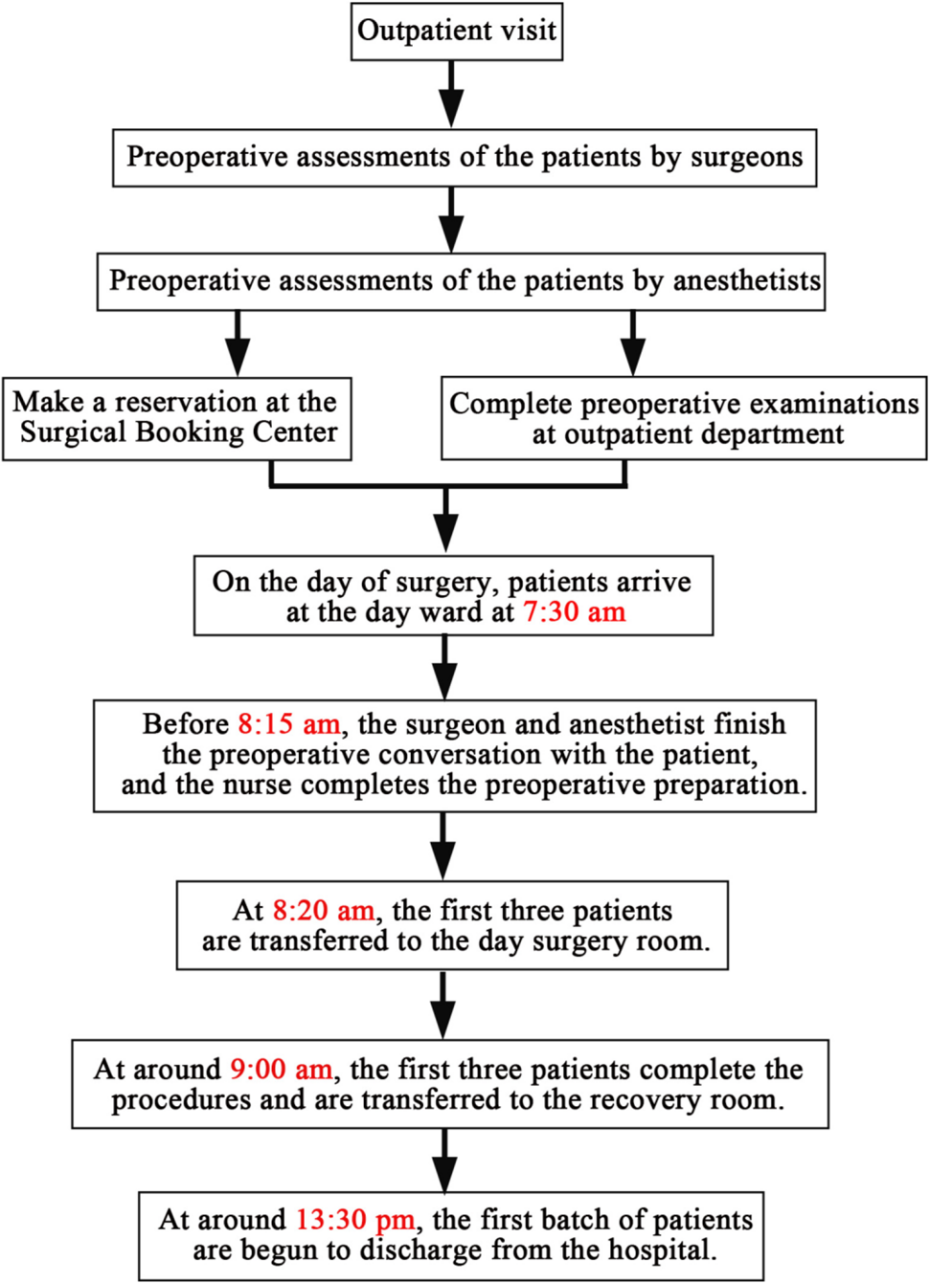
Process of day ward inpatient hospitalization

Surgeons and anesthesiologists perform preoperative assessments on patients in the outpatient department. Patients with admission certificates schedule their surgeries at the Surgical Booking Center and complete all required preoperative examinations prior to admission. On the day before admission, patients follow the fasting and water restriction instructions at the specified times. On the day of surgery, patients arrive at the day ward at 7:30 am and complete the admission procedures. Before 8:15 am, the surgeon and anesthesiologist finish the preoperative conversation with the patient’s parents, and the nurse completes the preoperative preparation. At 8:20 am, the first three patients are transferred to the day surgery room for laryngeal mask airway anesthesia. At approximately 9:00 am, the first three patients complete the procedures and are transferred to recovery room. The first batch of patients is discharged from the hospital at approximately 13:30 am if there are no special conditions (Fig. 2).

### Surgical procedures in LIHR

**Fig. 3:**
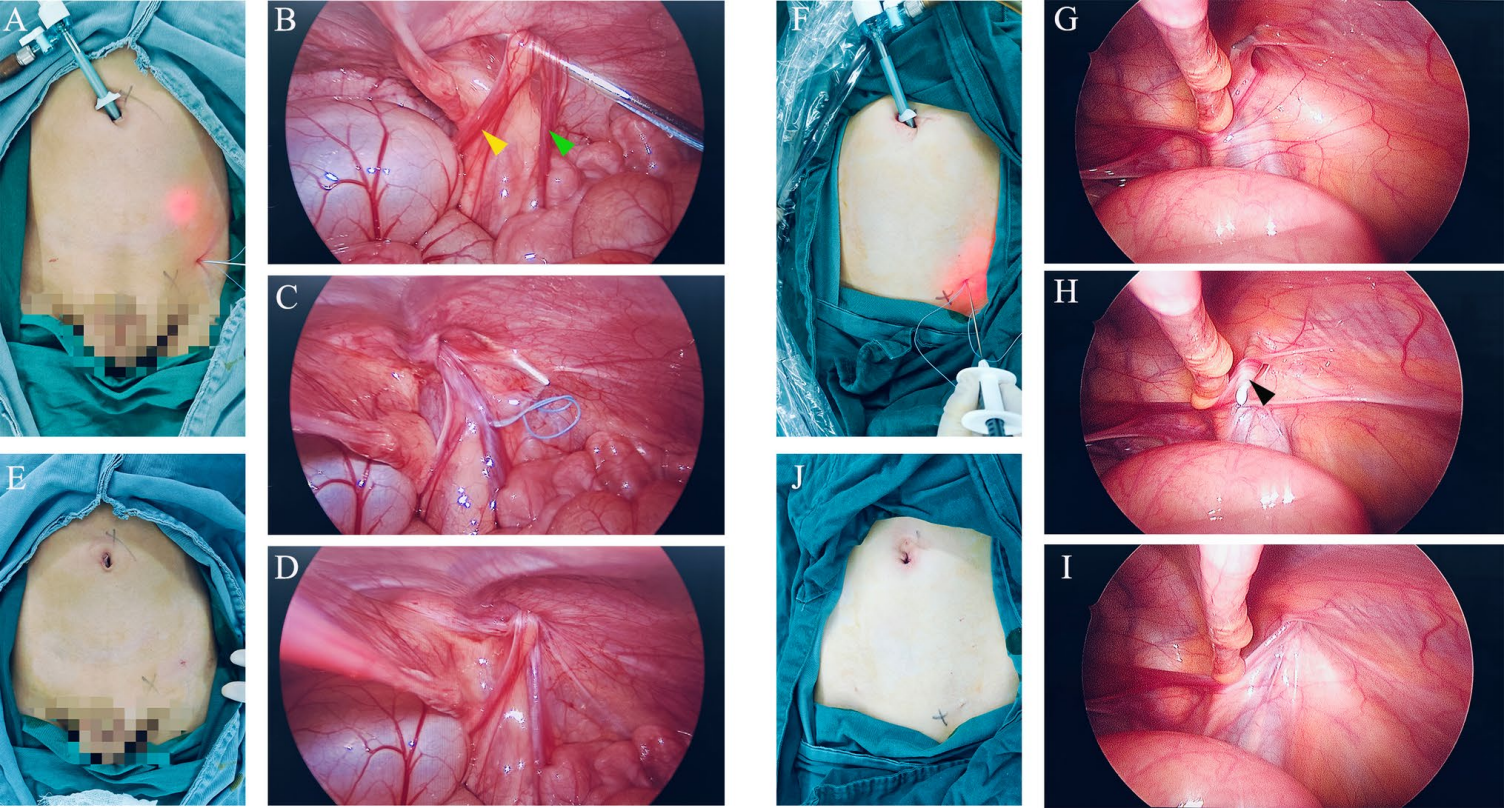
Surgical procedures for boy and girl with inguinal hernia. Surgical procedures for males: **A** Make a 5 mm incision beside the umbilicus and insert a 5 mm trocar as the main laparoscope, then locate the surface projection of the internal inguinal ring; **B** and **C** Use a hernia needle to encircle the internal inguinal ring across the vas deferens (yellow arrow) and spermatic cord vessels (green arrow); **D** Ligate the internal inguinal ring firmly; **E** Withdraw the trocar and suture the incision beside the umbilicus (Fig. 3**A**–**E**). Surgical procedures for females: **F** Make a 5-mm incision beside the umbilicus and insert a 5-mm trocar as the main laparoscope, then locate the surface projection of the internal inguinal ring; **G** Use a hernia needle to encircle the internal inguinal ring; **H** and **I** Ligate the internal inguinal ring firmly, and the round ligament of uterus also can be ligated; **J** Withdraw the trocar and suture the incision beside the umbilicus

Surgical procedures for males are as follows: 1) Make a 5 mm incision beside the umbilicus, insert a 5 mm trocar as the main laparoscope; 2) Locate the surface projection of the internal inguinal ring; 3) Use a hernia needle to encircle the internal inguinal ring across the vas deferens and spermatic cord vessels and ligate it firmly; 4) Withdraw the trocar and suture the incision beside the umbilicus (Fig. 3A–E).

Surgical procedures for females are as follows: 1) Make a 5 mm incision beside the umbilicus, insert a 5 mm trocar as the main laparoscope; 2) Locate the surface projection of the internal inguinal ring; 3) Use a hernia needle to encircle the internal inguinal ring and ligate it firmly, and the round ligament of uterus also can be ligated; 4) Withdraw the trocar and suture the incision beside the umbilicus (Fig. 3F–J).

### Follow-up and parent satisfaction survey

Patients discharged from the traditional and day wards are scheduled for outpatient follow-up on the 7th and 30th day postoperative days. Meanwhile, patients discharged from the day ward receive a telephone follow-up on the first day after hospital discharge. A satisfaction survey was administered via WeChat to 100 randomly selected parents from both the traditional and day wards. The questionnaire primarily focused on the following eight aspects: admission and discharge process; ward environment; surgical satisfaction; anesthesia satisfaction; nursing satisfaction; fasting management; postoperative health guidance; and hospitalization cost. Responses were divided into four levels: very satisfied (10 points), satisfied (8 points), average (6 points), and dissatisfied (4 points); with a total score of 80 points.

### Statistical analysis

All data were tested for normally distributed by Kolmogorov–Smirnov Test, and data were shown as the medians ± interquartile ranges (IQRs). Statistical analysis was performed with SPSS18.0 and GraphPad Prism 6. Categorical variables were compared using the Chi-square test, while independent samples t-tests were used for continuous, normally distributed variables. A p-value < 0.05 was considered statistically significant.

## Results

### OIHR and LIHR in traditional ward

A total of 8808 pediatric patients with IH were admitted to the Department of General Surgery ward. Of whom, 411 underwent OIHR (A1) and 8397 underwent LIHR (A2). Group A1 included 327 males and 84 females, with an average age of 2 years (IQRs, 1–5 years) and an average hospital stay of 2 days (IQRs, 2–4 days). Among the 411 patients in group A1, 272 had unilateral IH and 139 had a bilateral IH. The average hospitalization cost was 5840.82 RMB (range, 4700.60—7703.57 RMB). The postoperative hernia recurrence rate was 8.76% and 3 patients had incision infections. Group A2 consisted of 6388 males and 2009 females with an average age of 3 years (IQRs, 1–5 years) and an average hospital stay of 2 days (IQRs, 2–3 days). Of the 8397 patients in A2, 3964 had a unilateral IH and 4433 had a bilateral IH. The average operative time was 16.0 min (IQRs, 14.5–19.3 min), with a total time (operative time plus anesthesia time) was 49.0 min (IQRs, 46.0–53.8 min). The average hospitalization cost was 8353.39 RMB (IQRs, 7541.92–9367.51 RMB). The postoperative hernia recurrence rate was 0.12% and 7 patients had incision infections. Although the hospitalization cost for LIHR was significantly higher than for OIHR (p < 0.0001), the hernia recurrence rate was significantly lower in the LIHR group (p < 0.0001) (Table 1 and Table 2).

**Table 1:** Clinical characteristics of pediatric patients in day ward and department of general surgery ward

	A1: Department of general surgery ward (OIHR, n = 411)	A2: Department of general surgery ward (LIHR, n = 8397)	B1: Day ward (OIHR, n = 3776)	B2: Day ward (LIHR, n = 4965)	P (A1 vs. B1)	P (A2 vs. B2)
Sex (male: female)	327: 84	6388: 2009	2855: 921	3768: 1197	0.075	0.810
Age (year)	2 (1–5)	3 (1–5)	2 (1–4)	3 (2–5)	0.8715	P < 0.0001
Length of hospital stay (day)	2 (2–4)	2 (2–3)	1	1	/	/
Unilateral: bilateral	272: 139	3964: 4433	3318: 457	2317: 2648	/	0.545

*OIHR* open inguinal hernia repair, *LIHR* laparoscopic inguinal hernia repair

**Table 2:** Surgery-related data of pediatric patients in day ward and department of general surgery ward

	A1: Department of general surgery ward (OIHR, n = 411)	A2: Department of general surgery ward (LIHR, n = 8397)	B1: Day ward (OIHR, n = 3776)	B2: Day ward (LIHR, n = 4965)	P (A1 vs. B1)	P (A2 vs. B2)	P (B1 vs. B2)
Operative time (min)	/	16.0 (14.5–19.3)	/	14.5 (11.0–16.3)	/	0.1204	/
Total time (min)	/	49.0 (46.0–53.8)	/	37.0 (32.5–42.8)	/	P < 0.0001	/
Hospitalization cost (RMB)	5840.82 (4700.60- 7703.57)	8353.39 (7541.92–9367.51)	3614.51 (3425.07–3793.47)	6787.34 (6265.97–7281.24)	P < 0.0001	P < 0.0001	P < 0.0001
Incision infection (n)	3	7	8	4	P = 0.085	P = 1.000	P = 0.101
Recurrence rate (%)	36 (8.76%)	10 (0.12%)	94 (2.49%)	6 (0.12%)	P < 0.0001	P = 0.977	P < 0.0001

*Total time* operative time plus anesthesia time, *OIHR* open inguinal hernia repair, *LIHR* laparoscopic inguinal hernia repair

### OIHR and LIHR in day ward

A total of 8741 pediatric patients with IHs were admitted to the day ward, with 3776 underwent OIHR (B1) and 4965 underwent LIHR (B2). Group B1 included 2855 males and 921 females with an average age of 2 years (IQRs, 1–4 years). Among the 3776 patients, 3318 had a unilateral IH and 457 had a bilateral IH. The average hospitalization cost was 3614.51 RMB (IQRs, 3425.07–3793.47 RMB). The postoperative hernia recurrence rate was 2.49% and eight patients had incision infections. Group B2 was comprised of 3768 males and 1197 females with an average age of 3 years (IQRs, 2–5 years). Among the 4965 patients, 2217 had a unilateral IH and 2748 had a bilateral IH. The average operative time was 14.5 min (IQRs, 11.0–16.3 min) and the total time was 37.0 min (IQRs, 32.5–42.8 min). The average hospitalization cost was 6787.34 RMB (IQRs, 6265.97–7281.24 RMB). The postoperative hernia recurrence rate was 0.12% and four patients had incision infections. The hospitalization cost in the day ward were significantly lower than the traditional ward (p < 0.0001). Similarly, the hospitalization cost for laparoscopic surgery in the day ward was significantly higher than open surgery (p < 0.0001), but the hernia recurrence rate was significantly lower than open surgery (p < 0.0001). In addition, there were no significant differences with respect to gender, unilateral/bilateral cases, and operative time between patients who underwent LIHR in the day ward and the traditional ward. However, the total time in the day ward was significantly lower than the traditional ward (p < 0.0001). Parents of school-age children were more inclined to choose day surgery, resulting in a significantly higher average age for patients in the day ward compared to the traditional ward (p < 0.0001). Importantly, there were no significant differences in the rates of postoperative hernia recurrence and incision infections between group A2 and B2 (p = 0.977 and 1.000, respectively). However, the hospitalization costs in the day ward were significantly lower than the traditional ward (p < 0.0001) (Tables 1 and 2).

### Parent satisfaction survey results

The average parent satisfaction score in the day ward was 75.24 points (Table 3). Dissatisfaction primarily centered on the admission and discharge processes and anesthesia. Concerns about the admission and discharge process were noted when 78 individuals, including one patient and two parents per bed (26 beds), entered the ward simultaneously, leading to noise-related complaints. With respect to anesthesia, parents had concerns about the safety issues after discharge from the hospital because children were only observed for 4 h after anesthesia awakening. The average score for parent satisfaction in the traditional ward was 73.58 points (Table 4). The main areas of dissatisfaction were fasting management and the ward environment. Extended fasting times, due to lengthy preparation between surgeries, were a significant concern. Additionally, the traditional ward, with most rooms accommodating 3–4 beds plus 1–2 additional beds in the gallery, coupled with a longer average hospital stay (at least 3 days), contributed to reduced satisfaction with the ward environment.

**Table 3:** Parent satisfaction survey in the day ward

Aspects of satisfaction	Very satisfied (10 points)	Satisfied (8 points)	Average (6 points)	Dissatisfied (4 points)	Average score (N = 100)
Admission and discharge process (n)	24	56	20	0	8.08 points
Ward environment (n)	89	11	0	0	9.78 points
Surgical satisfaction (n)	95	5	0	0	9.90 points
Anesthesia satisfaction (n)	34	38	28	0	8.12 points
Nursing satisfaction (n)	88	12	0	0	9.76 points
Fasting management (n)	85	15	0	0	9.70 points
Postoperative health guidance (n)	95	5	0	0	9.90 points
Hospitalization cost (n)	100	0	0	0	10.00 points
Total average score: 75.24

**Table 4:** Parent satisfaction survey in the traditional ward

Aspects of satisfaction	Very satisfied (10 points)	Satisfied (8 points)	Average (6 points)	Dissatisfied (4 points)	Average score (N = 100)
Admission and discharge process (n)	81	15	4	0	9.54 points
Ward environment (n)	21	34	55	0	8.12 points
Surgical satisfaction (n)	96	4	0	0	9.92 points
Anesthesia satisfaction (n)	79	15	6	0	9.46 points
Nursing satisfaction (n)	85	15	0	0	9.70 points
Fasting management (n)	15	56	29	0	7.72 points
Postoperative health guidance (n)	84	5	11	0	9.46 points
Hospitalization cost (n)	84	15	1	0	9.66 points
Total average score: 73.58					

## Discussion

Single-incision laparoscopic treatment of pediatric IH has gained wide acceptance in the field of pediatric surgery after more than a decade of clinical practice [10]. LIHR not only reduces the postoperative recurrence rate [2, 11, 12], but also allows exploration for a contralateral IH and minimization of surgical related secondary damage [6, 13, 14]. Previously reported, the overall hernia recurrence rate of laparoscopic repair has been reduced to less than 1% [15], ranging from 0.13% to 0.42% [16, 17]. Shalaby et al., even reported that the postoperative recurrence rate was as low as 0% among 230 patients [18]. In our center, the recurrence rate of LIHR from 2016 to 2018 was 0.51% (6/1168). In the past 5 years (2018–2023), the recurrence rate of LIHR in our center has been reduced to 0.12% (16/13362), significantly lower than the reported data.

Day surgery has evolved along with the development of minimally invasive surgery, anesthesia, and anesthesia recovery techniques. After over 100 years of development, the proportion of day surgery has exceeded 60% in many European and American countries [19]. Our center begins exploring day surgery mode for pediatric OIHR since January 2016, with rapid development from 2018. Based on the OIHR experience in the day ward, we extended the day surgery mode to LIHR since 2020. This study aims to conduct a comprehensive analysis of the hospitalization efficiency and utilization of medical resources, clinical outcomes and safety, and patient satisfaction in the day ward.

### Hospitalization efficiency and utilization of medical resources in day ward

By optimizing perioperative processes, including pre-completed preoperative examination, efficient patient’s transportation between recovery room and operating room, experienced surgical team and homogeneous anesthetic management, the day surgery mode greatly improves the hospitalization efficiency. More specifically, a single operating room in the day ward can perform a maximum of six LIHR surgeries in a morning (8:20 am–12:30 am) in our hospital, compared to three in the traditional ward. Discharge procedures for the first batch of patients are typically completed by 13:30–14:00 pm. Day surgery mode also increases use of medical resources, achieving high bed occupancy rates and reducing hospitalization cost. In our experience, bed occupancy rates in day ward can reach up to 150%, and the hospitalization costs were significantly lower than in traditional ward. The efficient day surgery mode is the first choice for most parents, especially those who are busy with work. And the excellent utilization rate of medical resources is a management model that more hospitals are willing to choose and vigorously develop.

### Clinical outcomes and safety

In our study, the control of post-operative hernia recurrence rate in day ward matched that of the traditional ward, with no significant increase in the incidence of postoperative incision infections. The majority of pediatric patients are discharged successfully. with only a small number of patients experience delayed discharge due to postoperative nausea and vomiting (PONV), a main factor for delayed discharge in day surgeries [19, 20]. Among 8741 patients, three patients with severe PONV were transferred to traditional ward for conservative treatment, and the most severely affected patient was discharged after seven days of conservative treatment. Importantly, no anesthesia or surgical safety issues that endangered patient lives occurred, owing to strict three admission criteria (procedure, patient and surgeon) and three evaluation standards (preoperative, anesthesia and discharge assessments) [21]. Outstanding surgical and anesthesia safety, as well as a postoperative complication control rate that is not inferior to the traditional hospitalization mode, will inevitably make more parents willing and assured to choose the day surgery model.

### Patient satisfaction

The parent satisfaction score in day ward is higher than the traditional ward, especially in the aspects of surgical satisfaction, postoperative health guidance and hospitalization cost. One key factor is the standardized management of a single condition, allowing most anesthesiologists to use laryngeal mask airways, which facilitate faster intubation and extubation [22]. This is one of the important reasons for the high efficiency in day ward. Additionally, the fasting time for patients has been shorten by admitting patients in batches (the first batch at 7:30 am, the second batch at 10:00 am). Overall hospital stays in the day ward is controlled at approximately five hours, enhancing the patient experience by accelerating the surgical process through optimized perioperative management. Day surgery can be summarized in three words: safe, reliable and economical. This is also the main plus point for the high satisfaction of day surgery.

The present study has some limitations: retrospective nature, single-center, selection bias due to non-randomization. However, as the national clinical research center for child health, our patients come from all over the country, somewhat mitigating the selection bias and eliminating the impact of regional and demographic characteristics on the statistical data. In future research, we try to collaborate with regional pediatric medical centers to conduct multi-center studies, further evaluating whether the day surgery model can be further promoted in regional hospitals, particularly in developing countries where disparities in medical levels are notably prominent. Additionally, our center is trying to standardize and enhance the efficiency of this day surgery model and promote it across the entire surgical departments, including Oncology, Urology, Oral Surgery, Pediatric Gynecology, Cardiac Surgery, Thoracic Surgery, Otolaryngology, Orthopedics, Neurological Surgery, Ophthalmology, and Plastic Surgery. The procedures such as ligation of patent ductus arteriosus, excision of osteochondroma, excision of ovarian tumors, incision for hymenal obstruction, and adenoid or tonsil ablation are all relatively new practices. In 2024, a total of 6849 day surgeries have been completed across these departments, accounting for 22% of the total surgical volume at our hospital, significantly alleviating the pressure on hospital bed occupancy.

## Conclusion

In summary, for pediatric patients with the same medical condition, factors such as high surgical volumes, experienced surgeons, short operative times, and high level of uniformity in minimally invasive surgical techniques render day surgery particularly suitable, especially for pediatric IHs. Based on the surgical practice in our hospital over the past five years, the day surgery model has been proven to be a safe and reliable surgical management model and this model will help alleviate the tight medical resources in some areas.

## References

[1] Wang D, Yang P, Yang L, Jin S, Tang X (2020) Comparison of laparoscopic percutaneous extraperitoneal closure and laparoscopic intracorporeal suture in pediatric hernia repair. J Pediatr Surg 56:1894–1899. 10.1016/j.jpedsurg.2020.11.022 33309301 10.1016/j.jpedsurg.2020.11.022

[2] Tsai YC, Wu CC, Yang SD (2010) Open versus minilaparoscopic herniorrhaphy for children: a prospective comparative trial with midterm follow-up evaluation. Surg Endosc 24:21–24. 10.1007/s00464-009-0645-6 19690916 10.1007/s00464-009-0645-6

[3] Endo M, Watanabe T, Nakano M, Yoshida F, Ukiyama E (2009) Laparoscopic completely extraperitoneal repair of inguinal hernia in children: a single-institute experience with 1257 repairs compared with cut-down herniorrhaphy. Surg Endosc 23:1706–1712. 10.1007/s00464-008-0300-7 19343444 10.1007/s00464-008-0300-7 PMC2710496

[4] Safa N, Le-Nguyen A, Gaffar R et al (2023) Open and laparoscopic inguinal hernia repair in children: a regional experience. J Pediatr Surg 58:146–152. 10.1016/j.jpedsurg.2022.09.023 37306366 10.1016/j.jpedsurg.2022.09.023

[5] Davies DARD (2020) The international pediatric endosurgery group evidence-based guideline on minimal access approaches to the operative management of inguinal hernia in children. J Laparoendosc Adv Surg Tech A 30:221–227. 10.1089/lap.2016.0453 28140751 10.1089/lap.2016.0453

[6] Lomanto D, Chen WT, Fuentes MB et al (2023) Mastering endo-laparoscopic and thoracoscopic surgery: ELSA manual. Springer Nature, Singapore

[7] Nicoll JH (1917) Points in the operative treatment of hare-lip and cleft palate. Glasgow Med J 87:16–18 30434456 PMC5912874

[8] Nicoll JH (1905) Case operated on for radical cure of inguinal Hernia. Glasgow Med J 64:241–248 30437841 PMC5959496

[9] Sj H, Cj L, Qian Y (2019) Application of enhanced recovery after surgery for infantile inguinal hernia undergoing ambulatory surgery. J Clin PedSur 18:261–266. 10.3969/j.issn.1671-6353.2019.04.003

[10] Yang C, Zhang H, Pu J, Mei H, Zheng L, Tong Q (2011) Laparoscopic vs open herniorrhaphy in the management of pediatric inguinal hernia: a systemic review and meta-analysis. J Pediatr Surg 46:1824–1834. 10.1016/j.jpedsurg.2011.04.001 21929997 10.1016/j.jpedsurg.2011.04.001

[11] Schier F (2006) Laparoscopic inguinal hernia repair-a prospective personal series of 542 children. J Pediatr Surg 41(6):1081–1084. 10.1016/j.jpedsurg.2006.02.028 16769338 10.1016/j.jpedsurg.2006.02.028

[12] Niyogi A, Tahim AS, Sherwood WJ et al (2010) A comparative study examining open inguinal herniotomy with and without hernioscopy to laparoscopic inguinal hernia repair in a pediatric population. Pediatr Surg Int 26:387–392. 10.1007/s00383-010-2549-x 20143077 10.1007/s00383-010-2549-x

[13] Esposito C et al (2013) Laparoscopic repair of incarcerated inguinal hernia. A safe and effective procedure to adopt in children. Hernia 17:235–239. 10.1007/s10029-012-0948-8 22772871 10.1007/s10029-012-0948-8

[14] Harz M (2010) Surgical repair of incarcerated inguinal hernia in children: laparoscopic or open? Eur J Pediatr Surg 21:8–11. 10.1055/s-0030-1262793 20938898 10.1055/s-0030-1262793

[15] Li B, Nie X, Xie H, Gong D (2012) Modified single-port laparoscopic herniorrhaphy for pediatric inguinal hernias: based on 1,107 cases in China. Surg Endosc 26:3663–3668. 10.1007/s00464-012-2396-z 22773230 10.1007/s00464-012-2396-z

[16] Koivusalo AI, Korpela R, Wirtavuori K, Piiparinen S, Rintala RJ, Pakarinen MP (2009) A single-blinded, randomized comparison of laparoscopic versus open hernia repair in children. Pediatrics 123:332–337. 10.1542/peds.2007-3752 19117900 10.1542/peds.2007-3752

[17] Yonggang H, Changfu Q, Ping W et al (2019) Single-port laparoscopic percutaneous extraperitoneal closure of inguinal hernia using “two-hooked” core needle apparatus in children. Hernia 23:1267–1273. 10.1007/s10029-019-01933-9 30993474 10.1007/s10029-019-01933-9

[18] Shalaby R, Abdelmaboud M, Daboos M, Mohamed Y, Helal AA, Gamman I (2023) Needlescopic sutureless repair of congenital inguinal hernia: a randomized controlled study. Updat Surg 75:2327–2333. 10.1007/s13304-023-01566-9 10.1007/s13304-023-01566-9 PMC10710381 37341905

[19] De Luca U, Mangia G, Tesoro S, Martino A, Sammartino M, Calisti A (2018) Guidelines on pediatric day surgery of the Italian societies of pediatric surgery (SICP) and pediatric anesthesiology (SARNePI). Ital J Pediatr 44:35. 10.1186/s13052-018-0473-1 29530049 10.1186/s13052-018-0473-1 PMC5848546

[20] Bourdaud N, Devys J-M, Bientz J et al (2014) Development and validation of a risk score to predict the probability of postoperative vomiting in pediatric patients: the VPOP score. Paediatr Anaesth 24:945–952. 10.1111/pan.12428 24823626 10.1111/pan.12428

[21] Endoscopic Surgery Group CSOP (2020) Expert consensus on pediatric ambulatory surgery. Chin J Pediatr Surg 41:676–682. 10.3760/cma.j.cn421158-20200221-00106

[22] Garcia-Fernandez J, Tusman G, Suarez-Sipmann F, Llorens J, Soro M, Belda JF (2007) Programming pressure support ventilation in pediatric patients in ambulatory surgery with a laryngeal mask airway. Anesth Analg 105:1585. 10.1213/01.ane.0000287674.64086.f1 18042854 10.1213/01.ane.0000287674.64086.f1

